# Instagram as a Window to Societal Perspective on Mental Health, Gender, and Race: Observational Pilot Study

**DOI:** 10.2196/19171

**Published:** 2020-10-27

**Authors:** Kierstin Utter, Eva Waineo, Capricia M Bell, Harrison L Quaal, Diane L Levine

**Affiliations:** 1 Department of Internal Medicine Wayne State University School of Medicine Detroit, MI United States

**Keywords:** mental health, Instagram, social media, stigma, gender, race, depression

## Abstract

**Background:**

Gender and race are known to impact attitudes toward mental health topics and help-seeking behavior. Men and minorities are more likely to cite stigma as a reason for not seeking help for mental health concerns, which is of particular relevance given the high rate of suicide in men and challenges of historic proportion currently facing minority communities. Instagram provides a platform to discuss mental health, though a lack of male and minority representation may further alienate these populations.

**Objective:**

We aimed to investigate whether men and nonwhite individuals are underrepresented in Instagram photos tagged with #mentalhealth (compared to photos tagged with #health) to better understand how gender and race-based representations are manifested on this popular social media platform and discuss the implications.

**Methods:**

Three investigators of different genders and racial backgrounds met on nine different days via teleconference to analyze a total of 215 publicly available Instagram photos tagged with #mentalhealth and 215 with #health. These photos were generated using Instagram’s search function, and search results were sorted by most recently published at the time of data collection. For each photo, the three investigators recorded their observations about the gender (male versus female) and race (white versus nonwhite versus racially unclassifiable) of subjects featured in the photo, which they did not discuss with other investigators. Chi-squared analysis was performed on each investigator’s data set to compare the frequency of male versus female and white versus nonwhite subjects identified in each hashtag category. Kappa interrater agreement was calculated for each investigator pair, category (gender or race), and hashtag.

**Results:**

All three investigators observed significantly more female as compared to male subjects in photos tagged with #mentalhealth (*X*^2^=14.4, *P<*.001 for all investigators) while observing no significant difference between numbers of male and female subjects in photos tagged with #health (*X*^2^=1.533, *P*=.22; *X*^2^=1.241, *P*=.27; *X*^2^=0.096, *P*=.76). All three investigators identified significantly more white than nonwhite subjects in photos tagged with both #health and #mentalhealth (*X*^2^ values range from 11.912 to 98.927, *P*<.001 for all). Kappa interrater agreement revealed almost perfect agreement for gender (kappa=0.908-0.992) with the agreement for race ranging from 0.614 to 0.822, depending on hashtag and rater pair.

**Conclusions:**

Women are featured more frequently than men in Instagram photos tagged with #mentalhealth. The topic of #health, meanwhile, is not gendered this way. Low visibility of mental health among men may both represent and exacerbate existing stigma and barriers to care. White subjects are featured significantly more frequently than nonwhite subjects in photos tagged with both #mentalhealth and #health. Directed interventions using the Instagram platform may be indicated to increase the visibility of underrepresented groups and break the cycle of stigma.

## Introduction

With over 1 billion users [1], Instagram is one of the most widely used social media platforms in the world. It is, first and foremost, a visual platform, centered around sharing user-generated photos and videos accompanied by captions. Instagram allows users to search for photos by “hashtag.” A hashtag is a word or phrase preceded by the pound (#) symbol, which, when typed in the caption of the photo, makes the photo searchable by that term. Importantly, Instagram users have the option to make their photos private or public. Public photos are visible to anyone—even those without an Instagram account. Without an Instagram account, site visitors can only scroll through approximately 30 photos before either logging in or refreshing the page and starting anew. Visitors without accounts are likewise prohibited from clicking on photos returned in a search to access poster usernames or photo captions.

In addition to its popularity for social networking, Instagram is a growing forum for discussing health-related topics. The medical community has become interested in these discussions, such as those regarding HIV, cancer, vaping, alcohol, and self-harm behavior [2-6]. As of September 2020, there are 20.7 million posts tagged with #mentalhealth on Instagram and 123 million tagged with #health [7,8]. Instagram provides insight into the beliefs and attitudes circulating amongst users and allows health care providers to understand the social influence on health-related behavior.

We aimed to investigate whether men and nonwhite individuals are underrepresented in Instagram photos tagged with #mentalhealth (versus photos tagged with #health) to understand better how gender and race-based representations present on this popular social media platform and to discuss the implications. The rationale for this project stems from decades of research—on gender, race, and mental health stigma—and is twofold. We are interested first in whether historical patterns of gender and race-based stigma make themselves manifest in the influential social media realm, and in anticipating potential consequences of underrepresentation on this platform in particular.

Several risk factors known to be positively associated with symptoms of depression and anxiety are experienced more frequently by racial minorities, chief among which is discrimination [9]. Race also affects the rate at which patients receive treatment for mental illness, compounding these risks. Compared to 49.1% of non-Hispanic white patients that receive treatment, African American or non-Hispanic Black patients are treated at a rate of 30.6%, Hispanic or Latino patients 32.9%, and non-Hispanic Asian patients 24.9% [10]. Many implicate greater stigma in treatment discrepancies, especially among Asian and African American individuals. For example, in a study of attitudes toward mental health treatment in a college-aged student population, 63% of African American students and 52% of white students perceived stigma toward seeking care [11]. These figures are even more concerning in the current global climate when minority communities face a disproportionate death toll from the COVID-19 pandemic [12] and are leading a historic civil rights movement that brings intense focus to past personal traumas and injustice [13]. All these factors have created a sense of urgency in supporting the mental health of Black communities.

Mental health stigma is of particular relevance to men as well. A large historical body of research on gender, stigma, and mental health has shown that men associate mental illness and help-seeking with a deviation from the masculine ideal [14]. The development of this phenomenon may date back to the mid-1900s when the word “depression” first appeared in the Diagnostic and Statistical Manual [14]. It has been noted that male patients were “conspicuously absent” from the process of developing diagnostic criteria for depression and that early antidepressant clinical trials focused largely on women [14]. As a result, the tearful female patient became almost synonymous with depression [14].

The idea of depression and other mental health concerns as a “woman’s problem” is alive and well today. The most troubling evidence that diagnostic criteria may overlook men is the high rate of suicide in men despite a low rate of depression [15]. It is thought that men cover up emotional turmoil for fear of stigma; men are less likely than women to report symptoms of mood disorders, and when they do, they report their symptoms as less severe compared to women’s self-reports [15,16]. Additionally, men are more likely than women to list stigma as a reason for not seeking care [17]. Relying on negative male stereotypes to explain a lack of help-seeking behavior creates what some have described as a culture of “victim-blaming” [15]. Victim-blaming ignores cultural, societal, economic, and personal factors that may in fact play a large role in influencing men’s behavior around mental health, including norms set by society, peers, and today, social media.

Given its ubiquity, social media may affect users’ beliefs and attitudes toward mental health, and because it is a visual platform, gender and race feature prominently in Instagram posts. The gender and race of individuals in photos may influence how viewers feel about certain topics. For example, the gender and race of individuals in photos tagged with #mentalhealth may influence user attitudes on mental health—their beliefs regarding who talks about it, and who cares about it. In a recent qualitative study about men’s discourse surrounding mental health, one participant expressed awareness that there is a lack of “manly men” discussing mental health problems in public, while women do so more freely, creating space for other women to do the same [14].

Described in this introduction is a vicious cycle; stigma may lead to underrepresentation on Instagram, and this underrepresentation may lead to even greater alienation of men from discussions about mental health unless directed interventions are made. With this background in mind, we first predicted that women would be featured more frequently than men in photos tagged with #mentalhealth. Second, we predicted that white subjects would be featured more frequently than nonwhite subjects in photos tagged with #mentalhealth. These hypotheses are based on the assumption that experiencing more stigma in the “real world” would make individuals less likely to associate themselves visually with the topic of mental health on social media.

The hashtag #mentalhealth was chosen as a topic that, unlike #depression or #anxiety, lacks epidemiologic bias. It is well established that more women are diagnosed with depression and anxiety than men [14]. Mental health is not a diagnosis but describes the totality of emotional, social, and psychological factors that impact behavior, and therefore as a concept, is superimposable on all individuals regardless of gender or race. #Mentalhealth was also chosen to facilitate simple control. Our analysis included an identical data collection process for photos tagged with #health. Because “mental health” falls under the umbrella of “health,” we considered the number of men and women in photos tagged with #health to more closely reflect baseline Instagram representation. We hypothesized that equal numbers of male and female and white and nonwhite subjects would be featured in photos tagged with #health.

## Methods

In planning this project, investigators established that in order to investigate the research question of whether men and minorities are underrepresented in photos tagged with #mentalhealth as compared to #health, they would first have to generate a large pool of Instagram photos for each of these hashtags. The investigators would then look together at each photo, and each investigator would record the gender(s) and race(s) of subjects in these photos based on individual investigator’s interpretations. Because investigators predicted that they would disagree on gender or race for some subjects, data would need to be collected so that kappa agreement scores could be calculated.

This plan came to fruition in a multistep process with three investigators of different genders and racial backgrounds involved in data collection. The investigators met on nine separate days in July 2020 via teleconference. One investigator shared their screen and performed two internet searches in two separate tabs: one for “#mentalhealth Instagram” and another for “#health.” This search returned the respective links to access Instagram.com galleries of photos tagged with #mentalhealth and #health. Notably, the investigator was not logged into Instagram, such that only publicly available photos were visible. For both groups, photos were sorted such that the most recently posted appeared first, to avoid the bias of seeing the most “popular” photos. Photos featuring real human subjects and not meeting exclusion criteria were included for analysis. A detailed description of exclusion criteria is outlined in Multimedia Appendix 1.

One by one, eligible images were assigned a unique image ID, and investigators individually recorded the number of males, females, white, nonwhite, and racially unclassifiable subjects in each photo in separate excel sheets. During this process, investigators would be prompted to log in to Instagram, at which point the team would switch to data collection for the opposite hashtag after refreshing the page—this method avoids the need to log in. The popularity of these hashtags allowed this process to continue in a back-and-forth fashion, as dozens of new photos were added to each collection every few minutes.

On the first 8 data collection days, investigators recorded 25 photos from each hashtag. On the ninth data collection day, an additional 15 were added in each category if any data had to be removed after data collection. Investigators did not share their opinions about the gender or race of individuals in photos and did not share their data spreadsheets until data collection was complete.

Throughout this process, the authors used the term “gender” to describe the expressed gender identity of individuals in Instagram photos as investigators perceive it when forced to categorize as male or female. The term “white” was used among investigators to refer to individuals appearing to be of European descent. The term “nonwhite” refers to any individuals not meeting this definition, including Native and Indigenous populations.

Chi-squared analyses were used to determine the significance of differences between the total number of men versus women and white versus nonwhite subjects featured in each hashtag category. Investigators decided to analyze a minimum of 200 photos for each hashtag to have an 80% power to determine a 12% difference with alpha .05 or *P* value. In calculating chi-square analyses for race, individuals deemed to be “unclassifiable race” were excluded such that only a white versus nonwhite comparison was performed. Cohen kappa was used to calculate agreement between each pair of raters for each type of rating (gender or race) in each hashtag. Racially unclassifiable individuals were removed from this analysis, such that agreement focused on subjects whom raters deemed to be white or nonwhite. Chi-square values, *P* values, and kappa agreement scores were calculated using GraphPad Prism.

Out of respect for Instagram user privacy, included photos were not clicked on to reveal usernames or any other information about the posters. Only publicly available photos were included, and photos were not saved in any way. No identifying information was recorded or collected from individuals featured in photos. No one was contacted, and informed consent was waived. This research was exempted by the Institutional Review Board of Wayne State University (protocol #083519B3X).

## Results

Following data collection, ten mental health photos and six health photos were removed from all three investigators’ data sets due to transcription errors committed by at least one rater. For example, one rater may have marked a subject as both white and nonwhite, forgot to record gender, or missed a subject in a photo altogether. In order to calculate kappa scores accurately, these photos were removed from data analysis. As such, a total of 205 mental health photos and 209 health photos were analyzed. Raters categorized a total of 250 subjects in mental health photos and 261 subjects in health photos. Total numbers of males, females, white, nonwhite, and racially unclassifiable subjects observed by each investigator for #mentalhealth and #health are shown in Tables 1 and 2.

**Table 1:** Data summary by rater for #mentalhealth photos.

Rater	A	B	C
Female, n	155	155	155
Male, n	95	95	95
White, n	154	171	140
Nonwhite, n	96	73	87
Unclassifiable, n	0	6	23

**Table 2:** Data summary by rater for #health photos.

Rater	A	B	C
Female, n	141	140	133
Male, n	120	121	128
White, n	177	201	170
Nonwhite, n	83	45	59
Unclassifiable, n	1	15	32

### Rater A

In 205 photos tagged with #mentalhealth, rater A identified 155 females and 95 males and 154 white and 96 nonwhite individuals. In 209 photos tagged with #health, rater A identified 141 females and 120 males, and 177 white subjects, 83 nonwhite subjects, and 1 subject of unknown race.

### Rater B

In 205 photos tagged with #mentalhealth, rater B identified 155 females and 95 males, 171 white subjects, 73 nonwhite subjects, and 6 subjects of unknown race. In 209 photos tagged with #health, rater B identified 140 females, 121 males, 201 white subjects, 45 nonwhite subjects, and 15 subjects of unknown race.

### Rater C

In 205 photos tagged with #mentalhealth, rater C identified 155 females, 95 males, 140 white subjects, 87 nonwhite subjects, and 23 subjects of unknown race. In 209 photos tagged with #health, rater C identified 133 females, 128 males, 170 white subjects, 59 nonwhite subjects, and 32 subjects of unknown race.

All three investigators identified significantly more females than males in photos tagged with #mentalhealth while observing no significant difference between genders in photos tagged with #health (Table 3). All three investigators identified significantly more white than nonwhite individuals in photos tagged with both #mentalhealth and #health after removing individuals of unknown race.

**Table 3:** Chi-square analyses by rater, comparison, and hashtag.

Rater	Comparison	Hashtag	Chi square	*P* value
A	F^a^ vs M^b^	MH^c^	14.4	<.001
A	W^d^ vs NW^e^	MH	13.456	<.001
A	F vs M	H^f^	1.533	.22
A	W vs NW	H	33.985	<.001
B	F vs M	MH	14.4	<.001
B	W vs NW	MH	39.361	<.001
B	F vs M	H	1.241	.27
B	W vs NW	H	98.927	<.001
C	F vs M	MH	14.1	<.001
C	W vs NW	MH	11.912	<.001
C	F vs M	H	0.096	.76
C	W vs NW	H	53.803	<.001

^a^F: female

^b^M: male

^c^MH: #mentalhealth

^d^W: white

^d^NW: nonwhite

^e^H: #health

Interrater agreement for gender across both hashtags ranged between 0.908 and 0.992, representing almost perfect agreement (Table 4). Interrater agreement for race in #mentalhealth photos ranged from 0.784 to 0.822. Interrater agreement for race in #health photos was the lowest, ranging from 0.614 to 0.688.

**Table 4:** Kappa interrater agreement by rater pair, category, and hashtag.

Rater 1	Rater 2	Category	Hashtag	Kappa	
A	C	Gender	MH^a^	0.983	
A	B	Gender	MH	0.949	
C	B	Gender	MH	0.966	
A	C	Race	MH	0.822	
A	B	Race	MH	0.784	
C	B	Race	MH	0.805	
A	C	Gender	H^b^	0.908	
A	B	Gender	H	0.992	
C	B	Gender	H	0.916	
A	C	Race	H	0.688	
A	B	Race	H	0.662	
C	B	Race	H	0.614	

^a^MH: #mentalhealth

^b^H: #health

## Discussion

### Principal Findings

Our study highlights several important points about the visual representation of #mentalhealth on Instagram. First, females are represented significantly more often than males. This difference is not seen in photos tagged only with #health. Therefore, we believe the gender difference observed in #mentalhealth photos is not simply due to greater Instagram usage by women but is an extension of the long-observed tendency for men to withhold from public discussions of mental health [14-17].

It is thought that this tendency is motivated by stigma and the desire to conform to the hegemonic masculine ideal, in which men are expected to hide emotional turmoil and only seek help after experiencing significant pain or physical injury [14]. Much of the work on this topic has focused on men and topics with negative or health-impairing connotations, such as depression and anxiety [14,15,18]. Our study focused on mental health generally—a phrase that may arise in discussions about depression but may also arise in discussions of happiness, recovery, or wellness. Real-world stigma is, therefore, not only replicated in the social media realm but perhaps exposed even further; it is possible that beyond mental illness and help-seeking, men also face barriers when it comes to discussing wellness.

Perhaps the most troubling interpretation of our data is that social media may propagate the idea of “mental health” as a woman’s cause. Previous research on stigma and men’s mental health has demonstrated that when certain health behaviors, such as health care utilization, are performed more frequently by women, men associate these behaviors with deviation from the masculine ideal and become stigmatized [19]. Discussion of mental health topics on social media may play out similarly. Unfortunately, what results is a self-propagating cycle in which stigma may decrease the visibility of men’s mental health, which intensifies the stigma.

Our results also highlighted a disproportionate representation of white individuals in photos in both #health and #mentalhealth categories, representing a departure from our hypothesis. We expected to see more white and nonwhite subjects in #mentalhealth photos, but equal numbers of white and nonwhite subjects in #health photos. These results may reflect a real phenomenon of greater engagement with *both* health and mental health topics by white users. However, it is also important to consider that the majority (60.1%) of the US population is white [20].

### Future Interventions and Directions

Our findings suggest an opportunity for intervention using the Instagram platform. Disruption of the underrepresentation of men and minorities in conversations about mental health (and health, in the case of minorities) may slow the perpetuation of stigma by helping Instagram users see mental health as a cause for everyone. Interventions may take the form of Instagram campaigns designed to reach men and minorities, focusing on circulating images of individuals from these groups tagged with #mentalhealth and #health.

Intervention may also be warranted in the health care setting, as our observations underscore the importance of patient education and patient-centered care. That is, physicians have a unique opportunity to engage male and minority patients in discussions about their health that may be stigmatized or not discussed in their personal lives. Further, demonstrating an understanding that different demographic groups discuss health to varying degrees contributes to cultural competence and the delivery of empathic care.

The methods described here have the potential for reproducibility across a wide array of disciplines within and outside of medicine. Instagram as research tool is a relatively new concept, and methodology remains a challenge in this field. In a study published in the Journal of Medical Internet Research in 2017, Carrotte et al stated, “No best practice tools are available for systematically searching social media, and various websites’ default search algorithms do not allow systematic searching [21].” Though this remains true today, our methods are similar to those of previously published works in the field and are, in some ways, more rigorous.

Carrotte et al, for example, used two coders to analyze 476 social media posts from Instagram, Tumblr, Facebook, and Twitter tagged with #fitspo using the respective sites’ search engines [21]. Images were collected over ten minutes. These authors coded images on 28 different variables, and only included categorical variables in the analysis if the kappa agreement met a minimum value of .60. Tiggemann and Zaccardo used Instagram’s search function to identify photos tagged with #fitspiration [22]. One coder analyzed 600 images, assigning categorical variables to the image content such as food (healthy versus not healthy), gender (male, female, or both), and adiposity (thin, average, or overweight). A second independent coder assessed 10% of the included images. Santarossa et al used the Netlytic program to download Instagram photos tagged with #fitspo over 4 days [23]. This method yielded 128 photos coded by two investigators into photo categories (action, objectification, selfie, supplement, or other). Naftali et al used two coders (an experienced social media user and a social media expert) to analyze 300 photos from Facebook, Instagram, and YouTube, using the search term #plastic_surgery on Instagram [24]. Variables analyzed by coders included the poster’s identity, photo subject (self-promotional, educational, commercial, or personal), and whether the photo featured “shaming.”

Like our study, these works utilized hashtags to generate their database of photos and multiple human coders [21-24]. Our project’s methods are more robust in that three human coders were utilized, and data were collected over 2 weeks to create a more randomized data set. Moreover, our sample size falls within the range of all these published studies, and our kappa agreement scores all fall above .60, the acceptable minimum used by Carrotte et al [21].

We believe our work adds to research related to mental health representations in social media, but it is only a starting point. This methodology can be used to study other hashtags related to specific mental health illnesses (eg, depression or anxiety) and even hashtags related to recovery. Future studies may also examine what other hashtags are commonly associated with photos tagged with #mentalhealth that may provide further insight into posters’ attitudes toward mental health.

### Limitations

The findings outlined in this study, particularly as it pertains to gender, are reliable in that three different raters of different racial and gender backgrounds individually identified statistically significant patterns with almost perfect agreement. Agreement scores for race determination were lower than that for gender, suggesting that race was predictably more subjective than gender, perhaps due to individual bias regarding physical features and racial categories. Notably, because racially unclassifiable individuals were removed from this analysis, there were fewer data points used to calculate kappa scores for race as compared to gender.

As noted in the methods section, subjects deemed to be of “unclassifiable race” were also excluded from chi-squared calculations. Initially, there were concerns about the effect of excluding these individuals, as it could falsely inflate the representation of one race. However, despite recording different numbers of unclassifiable race subjects (see Tables 1 and 2), the three investigators observed the same patterns regarding gender and race discrepancy when the chi-squared analysis was performed (Table 3).

The social and political environment impacts the content of Instagram photos. To mitigate bias that may occur from collecting data at one specific time point coinciding with the circulation of a “viral” topic, investigators collected data on nine separate occasions over two weeks. Although photo inclusion and exclusion criteria were established at the time of data collection to focus the research question and prevent bias from the selection of photos, several photos meeting the inclusion criteria were noted to pose a challenge for raters (eg, taken from several meters away, in low lighting, or with faces/heads obstructed). Future studies may implement even stricter inclusion criteria to focus data collection even more closely.

The investigators are aware of a blind spot in this design in assessing the number of transgender, gender fluid, and non-binary individuals, and also of the fundamentally flawed nature of gender categorization based on physical appearance. However, this study is interested in binary categorization to explore the stigma resulting from a binary system. It is also possible that the individuals featured in photos were of different gender and race of the individual posting the photo, but for this study focusing on visibility, the focus remains on the photo itself. As a minor point, several video thumbnails were included in analyses. The videos may have featured individuals not seen in the thumbnail. Finally, hashtags were English words, and searching for photos with #health and #mentalhealth translated into other languages may impact data, particularly racial data.

### Conclusions

This observational pilot study found that women are featured more frequently than men in public Instagram photos tagged with #mentalhealth, while there is no significant difference in the number of men and women featured in photos tagged with #health. Past research suggests these findings may be due to hegemonic gender norms and stigma. White subjects appear more frequently than nonwhite subjects in photos tagged with both #health and #mentalhealth. These disparate findings lay bare the need to promote the visibility of underrepresented groups in discussions surrounding mental health on social media and provide an emerging platform for health care providers to do so.

## Appendix

Multimedia Appendix 1 Photo inclusion and exclusion criteria.

## Abbreviations

H: #health
MH: #mentalhealth
NW: nonwhite
W: white

## References

[1] Number of monthly active Instagram users from January 2013 to June 2018.

[2] Nobles AL, Leas EC, Latkin CA, Dredze M, Strathdee SA, Ayers JW (2020). #HIV: Alignment of HIV-Related Visual Content on Instagram with Public Health Priorities in the US. AIDS Behav.

[3] Basch C, MacLean S (2019). Breast Cancer on Instagram: A Descriptive Study. Int J Prev Med.

[4] Czaplicki L, Kostygina G, Kim Y, Perks SN, Szczypka G, Emery SL, Vallone D, Hair EC (2019). Characterising JUUL-related posts on Instagram. Tob Control.

[5] Hendriks H, Wilmsen D, van Dalen W, Gebhardt WA (2019). Picture Me Drinking: Alcohol-Related Posts by Instagram Influencers Popular Among Adolescents and Young Adults. Front Psychol.

[6] Arendt F (2019). Suicide on Instagram - Content Analysis of a German Suicide-Related Hashtag. Crisis.

[7] (2020). #mentalhealth. Instagram.

[8] (2020). #health. Instagram.

[9] Williams DR (2018). Stress and the Mental Health of Populations of Color: Advancing Our Understanding of Race-related Stressors. J Health Soc Behav.

[10] (2019). Mental Health By The Numbers. National Alliance on Mental Illness.

[11] Lipson SK, Kern A, Eisenberg D, Breland-Noble AM (2018). Mental Health Disparities Among College Students of Color. J Adolesc Health.

[12] Laurencin CT, McClinton A (2020). The COVID-19 Pandemic: a Call to Action to Identify and Address Racial and Ethnic Disparities. J Racial Ethn Health Disparities.

[13] Crowell C, Mosley D, Falconer J, Faloughi R, Singh A, Stevens-Watkins D, Cokley K (2017). Black Lives Matter: A Call to Action for Counseling Psychology Leaders. Couns Psychol.

[14] Johnson J, Oliffe J, Kelly M, Galdas P, Ogrodniczuk J (2012). Men's discourses of help-seeking in the context of depression. Sociol Health Illn.

[15] Affleck W, Carmichael V, Whitley R (2018). Men's Mental Health: Social Determinants and Implications for Services. Can J Psychiatry.

[16] Simon R (2014). Sociological Scholarship on Gender Differences in Emotion and Emotional Well-Being in the United States: A Snapshot of the Field. Emotion Review.

[17] Ojeda VD, Bergstresser SM (2008). Gender, race-ethnicity, and psychosocial barriers to mental health care: an examination of perceptions and attitudes among adults reporting unmet need. J Health Soc Behav.

[18] Smith DT, Mouzon DM, Elliott M (2018). Reviewing the Assumptions About Men's Mental Health: An Exploration of the Gender Binary. Am J Mens Health.

[19] Noone J, Stephens C (2008). Men, masculine identities, and health care utilization. Sociology of Health and Illness. (30).

[20] (2019). QuickFacts. United States Census Bureau.

[21] Carrotte ER, Prichard I, Lim MSC (2017). "Fitspiration" on Social Media: A Content Analysis of Gendered Images. J Med Internet Res.

[22] Tiggemann M, Zaccardo M (2018). Strong is the new skinny: A content analysis of #fitspiration images on Instagram. J Health Psychol.

[23] Santarossa S, Coyne P, Lisinski C, Woodruff SJ (2019). #fitspo on Instagram: A mixed-methods approach using Netlytic and photo analysis, uncovering the online discussion and author/image characteristics. J Health Psychol.

[24] Ben Naftali Y, Duek O, Rafaeli S, Ullmann Y (2018). Plastic Surgery Faces the Web. Plastic and Reconstructive Surgery - Global Open.

