# Supplementary material for: Instagram as a Window to Societal Perspective on Mental Health, Gender, and Race: Observational Pilot Study
Source: JMIR Ment Health. 2020 Oct 27;7(10):e19171. doi: 10.2196/19171 (PMC7655468; doi:10.2196/19171)
Supplement: Multimedia Appendix 1 [file mental_v7i10e19171_app1.docx]

##### Appendix 1

#### Photo Inclusion and Exclusion Criteria

Photos featuring real human subjects were considered for analysis. Exclusion criteria included:

- Cartoon or computer-generated depictions of humans.
- Photos featuring only hands or feet.
- Repeat photos (already analyzed as per the investigator’s memory).
- Silhouette photos.
- Photos of humans taken from a significant distance away such that anonymity was preserved.

Photo eligibility was agreed upon by all investigators for all photos.

#### Children

Photos featuring only children (agreed on by all three investigators to be under 18 years of age) were not included. Photos featuring both adults and children were included, however race and gender of children was not recorded. Investigators reasoned that children in photos are unlikely to be the individual who posted the photo, and agreed to narrow focus to the adult population.

#### Group photos

Photos of groups larger than 12 people were not included. This is primarily because the smaller size of all subjects led to anonymity similar to that of photos taken from a significant distance.

#### “Before and After” Photos

Photos composed of two panels depicting the same person (agreed upon by all three investigators) were counted as only 1 individual.
